# Psychological impact on care professionals due to the SARS‐Cov‐2 virus in Spain

**DOI:** 10.1111/inr.12748

**Published:** 2022-02-02

**Authors:** Iván Santolalla‐Arnedo, Pablo Del Pozo‐Herce, Regina Ruiz De Viñaspre‐Hernandez, Vicente Gea‐Caballero, Raúl Juarez‐Vela, Guadalupe Gil‐Fernandez, Rebeca Garrido‐Garcia, Emmanuel Echaniz‐Serrano, Michał Czapla, Francisco José Rodriguez‐Velasco

**Affiliations:** ^1^ Faculty of Nursing, Group of Research in Sustainability of the Health System Biomedical Research Center of La Rioja (CIBIR) University of La Rioja Logroño Spain; ^2^ Department of Psychiatry Fundación Jiménez Díaz University Hospital Madrid Spain; ^3^ Biomedical Research Center of La Rioja (CIBIR) Group of Research in Sustainability of the Health System Logroño Spain; ^4^ Faculty of Health Science International University of Valencia Valencia Spain; ^5^ Research Group PBM Patient Blood Management Health Research Institute IdiPAZ, Hospital La Paz Madrid Spain; ^6^ Department of Nursing Faculty of Medicine and Health Science University of Extremadura Extremadura Badajoz Spain; ^7^ Rioja Health Service Spain Group of Research in Sustainability of the Health System Biomedical Research Center of La Rioja (CIBIR) Najera Health Center, La Rioja Logroño Spain; ^8^ Department of Nursing and Physiatry Transfecult Research Group University of Zaragoza Zaragoza Spain; ^9^ Faculty of Health Sciences Wroclaw Medical University Wroclaw Poland; ^10^ Center for Heart Diseases University Hospital Worclaw Poland

**Keywords:** Coronavirus, COVID‐19, mental health, nursing, SARS‐CoV‐2

## Abstract

**Objective:**

To analyze the psychological impact of the SARS‐Cov‐2 pandemic on nurses in Spain in three different dimensions: exposure to stressors, perceived emotions, and stress coping.

**Background:**

On March 11, 2019, the World Health Organization recognized a global pandemic caused by a SARS‐Cov‐2 virus, COVID‐19, which rapidly spread across the planet, involving a community health emergency of international scope.

**Introduction:**

The pandemic situation in health centers has led to significant changes in the work environment, compromising care professionals’ physical and psychological health and resulting in strong physical and mental exhaustion.

**Methods:**

An observational, descriptive, cross‐sectional study was carried out, between February and April 2021, in a large sample of 1360 participants. The researchers conducted the dissemination of a validated questionnaire to working nurses in Spain.

**Results:**

The sex variable in relation to the study dimensions (stressors, perceived emotions, and coping strategies) showed a mean for stressors of 62.2 ± 10.5 in women and 59.8 ± 12.5 in men (p = 0.010), showing statistically significant differences. Age was a protective factor for all dimensions (p < 0.001). Time of experience showed statistically significant differences for stressors and coping strategies in professionals with more than 15 years of experience.

**Discussion:**

Female nurses who are younger, have less work experience, have not built a family of their own, and live in smaller or indoor flats may be more vulnerable to the effects of the COVID‐19 pandemic on their mental health. Other national and international studies, in this line, have shown an important psychological impact on these professionals.

**Conclusion:**

It is necessary to design and adopt effective strategies and measures for the protection of nurses' mental health, as well as for the prevention and early diagnosis of possible mental health problems.

## INTRODUCTION

On March 11, 2020 , the World Health Organization (WHO) recognized a global pandemic caused by a SARS‐Cov‐2 virus, COVID‐19, which rapidly spread across the planet, involving a community health emergency of international scope (Zhu et al., 2020). To date, 177 108 695 cases of COVID‐19 have been registered worldwide since the pandemic began in December 2019 in Wuhan, China (WHO, 2021).

The pandemic situation in health centers has led to significant changes in the work environment of health professionals, compromising their psychological and physical health and resulting in strong physical and mental exhaustion (Chew et al., 2020; Moreira et al., 2020; Reger et al., 2020; Torales et al., 2020). Health professionals have faced significant pressure, with long work shifts (Huarcaya‐Victoria, 2020), ethical dilemmas regarding the allocation of scarce resources to patients with the same needs, high risk of infection, inadequate working conditions (Ramírez‐Ortiz et al., 2020), negative emotions (Lin et al., 2020; Restauri & Sheridan, 2020; Sun et al., 2020; Tan et al., 2020), lack of specific skills, frustration (Paiano et al., 2020), social stigmatization, isolation, concern about infecting their families (Almaguer et al., 2020; Huarcaya‐Victoria, 2020), and lack social support (Lai et al., 2020).

These changes, and the context in which work has been carried out in health and social health centers in the last year, have given rise to increased psychological pressure, on health professionals, which can trigger feelings of helplessness, stress, loneliness (Liu et al., 2019; Ornell et al., 2020), anguish (Chew et al., 2020), depression (Huarcaya‐Victoria, 2020; Liu et al., 2019), anxiety (Paiano et al., 2020; Rajkumar, 2020), denial, anger, irritability, fear, sleep disorders (Liu et al., 2020b; Moreira et al., 2020), burnout (Ramírez‐Ortiz et al., 2020), and even risk of suicide (Reger et al., 2020). All this increases the probability of developing mental disorders (Ornell et al., 2020; Paiano et al., 2020; Torales et al., 2020), vicarious trauma related to compassion toward the patients attended (Li et al., 2020), or posttraumatic stress, which can even become chronic (Ramírez‐Ortiz et al., 2020; Vieta et al., 2020; Wand et al., 2020). The perceived stress may amplify the effects of an underlying diathesis, leading to the development of a mental disorder. The diathesis–stressor model may be a useful approach to determine how preexisting traits (diathesis) interact with the environmental surroundings (stressors) determining coronavirus‐related pathological responses (Cox et al., 2020).

Studies carried out during the pandemic have shown that more than 75% of health workers who are in high‐risk situations have developed psychological symptoms (Moreira et al., 2020; Paiano et al., 2020; Ramírez‐Ortiz et al., 2020). Half of these health workers reported anxiety and depressive symptoms, while more than 30% reported sleep disturbances (Chew et al., 2020; Huarcaya‐Victoria, 2020; Lai et al., 2020; Liu et al., 2019; Ramírez‐Ortiz et al., 2020; Reger et al., 2020).

The levels of anxiety and psychological pressure have been related to direct and continuous contact with patients infected by SARS‐CoV‐2 (Crowe et al., 2021; Liu et al., 2019), with higher levels being found in nurses than in medical personnel, since they generally have closer and longer contact with patients (Lin et al., 2020; Liu et al., 2019; Ornell et al., 2020; Tan et al., 2020).

The first step for effective management of the effect of the COVID‐19 pandemic on nurses' health is to have key, reliable, and updated information.

## AIM OF STUDY

The study's aim was to analyze the psychological impact of pandemic on nurses in Spain in three different dimensions: exposure to stressors, perceived emotions, and stress coping. This information would help design protocols and risk control procedures, promoting the acquisition of coping strategies.

## MATERIALS AND METHODS

### Study design

An observational, descriptive, cross‐sectional study was conducted between February and April 2021. The study complies with the STROBE checklist for cross‐sectional studies.

### Population and scope of the study

The study was carried out in all the autonomous communities of Spain. The questionnaire was distributed to registered nurses and nursing auxiliaries. Nonprobabilistic purposive sampling by the investigators was used. The results obtained during the questionnaire carried out between February 15 and April 15, 2021 were analyzed.

### Data collection instrument/procedure

Different scientific and professional entities assisted with the dissemination of a previously used and validated questionnaire. The questionnaire was based on the following three dimensions: first, exposure to stressors, there were items on the conditions occurring in the work environment that may affect their psychological state; second, emotions perceived in the workplace related to the pandemic state; and finally, coping strategies aimed at identifying how workers coped with this situation. Regarding the format of the survey, the questionnaire included questions on stressors, perceived emotions, and coping strategies, which were answered using a forced‐choice Likert‐type scale from 1 to 5 (1 strongly disagree and 5 strongly agree). A previous study (Del Pozo‐Herce et al., 2021) tested the psychometric proprieties of variable stressors, perceived emotions, and coping strategies used in this research with a Cronbach's alpha of 0.9, 0.8, and 0.8, respectively. The validation of the internal consistency of each item was rated “very good”; the alpha value was equal to or higher than 0.7, which allowed us to be confident in the results of the study, highlighting its robustness. The first dimension (stressors), consisting of 18 items, adopted values between 0 and 90; the second dimension with 4 items (perceived emotions) adopted values between 0 and 20; and the third dimension, also with 4 items (coping strategies), adopted values between 0 and 20.

### Study variables

Primary variables in the study population were associated with the dimensions, i.e., stressors, perceived emotions, and coping strategies. Secondary variables included demographic data of professionals (autonomous community, sex, age, marital status, dependents, number of children, type of housing, and cohabitants), and other data related to their jobs (professional category, years of professional experience, and type of contractual relationship with the company).

### Statistical procedures

Descriptive statistics (mean and standard deviation) were used to report quantitative values. The distribution of categorical variables is shown by absolute and relative frequencies. We described the main variables independently for each of the three dimensions of the questionnaire used and calculated the Cronbach's reliability coefficient alpha for each dimension.

Residuals of the models were tested randomly with a normal distribution of zero mean and constant variance. The “stressors” model has a normal distribution (Shapiro–Wilk test value for residuals = 0.235) of mean 0 and constant variance (p = 0.290, Breusch–Pagan heteroscedasticity test). F‐statistic (12,1347) = 10.50, p < 0.001. In the “perceived emotions” model, the distribution is normal (Shapiro–Wilk test value for residuals = 0.432) of mean 0 and constant variance (p = 0.085, Breusch–Pagan heteroscedasticity test). F‐statistic (7,1352) = 10.76, p < 0.001. The third model, “coping strategies,” is normally distributed (Shapiro–Wilk test value for residuals = 0.189) of mean 0 and constant variance (p = 0.739, Breusch–Pagan heteroscedasticity test). F‐statistic (7,1352) = 5.37, p < 0.001.

Univariate linear regression was performed combining each of the dimensions of the questionnaire with sociodemographic variables. The study was strengthened by calculating multiple linear regression by introducing each of the dimensions of the questionnaire and all the demographic variables that had been significant in the univariate regression as independent variables. The threshold for significance was set at a two‐sided alpha value of 0.05 for all analyses.

### Ethical considerations

This study was developed in accordance with the Data Protection Regulation (EU) 2016/679 of the European Parliament and the Organic Law 3/2018 on data protection. The information was treated confidentially and anonymously as the data were dissociated. The study was approved by the ethics committee of La Rioja CEimLAR (reference P.I. 416).

## RESULTS

### Sociodemographic characteristics

In total, 1360 healthcare professionals answered the questionnaire. Interestingly, 89% of the study participants were women. The mean age was 42.1 years, the minimum age was 18, and the maximum age was 70 years. The community of Madrid had the highest response rate with 25.3%, followed by La Rioja with 11%, and that of Valencia with 10%. The sample turned out to be equal in terms of the professional category, with 56.6% of registered nurses versus 43.4% of nursing auxiliaries. Table 1 shows complete sociodemographic data.

**TABLE 1:** Sociodemographic characteristics

Characteristics	*n*	%
Age		
<30	275	20.2
30−45	543	39.9
>45	542	39.9
Sex		
Male	151	11.1%
Female	1209	88.9%
Marital status		
Single	470	34.6%
Married	656	48.2%
Divorced	110	8.1%
Widower	20	1.5%
Other	104	7.6%
Dependents in my charge		
Yes	188	13.8%
No	1172	86.2%
Type of housing		
Interior flat	203	14.9%
Outside flat	815	59.9%
Terraced house with garden	177	13.1%
House with private land	165	12.1%
Living in company		
Yes	1142	84%
No	218	16%
Category		
University (nurse)	770	56.6%
Not university (nurse auxiliary)	590	43.4%

### Stressors, perceived emotions, and coping strategies

We can observe that the first dimension (stressors) takes a mean of 61.2 ± 10.7, with a minimum of 26 and a maximum of 94. The second dimension (perceived emotions) takes a mean of 14.7 ± 3.9, with a minimum of 4 and a maximum of 20. The third dimension (coping strategies) takes a mean of 10.6 ± 3.7, with a minimum of 4 and a maximum of 20 (Supporting Information S1). Cronbach's alpha coefficients for each dimension were 0.8 for stressors and perceived emotions and 0.7 for coping strategies.

Table 2 shows the scores obtained from the questionnaire (mean and standard deviation) for the independent variables, and Table 3 lists the results of univariate and multivariate regression between the independent (sex, age, civil status, type of living, type of employment relationship, and work experience) and dependent variables, i.e., the dimensions (stressors, perceived emotions, and coping strategies).

**TABLE 2:** Mean and standard deviation of the dimensions

	Stressors		Perceived emotions		Coping strategies	
	Mean	SD	Mean	SD	Mean	SD
Sex						
Male	59.8	± 12.5	13.6	± 4.4	10.1	± 4
Female	62.2	± 10.5	14.9	± 3.8	10.6	± 3.7
Age						
<30	64.9	± 9.7	15.7	± 3.4	11.2	± 3.5
30−45	63.2	± 10	14.9	± 3. 8	10.7	± 3.8
>45	59.3	± 10.8	14.1	± 4	10.2	± 3.6
Marital status						
Single	63.4	± 10.3	15.1	± 3.6	11.1	± 3.6
Married	61.6	± 10.6	14.4	± 3.9	10.3	± 3.7
Divorced	58.7	± 11.4	14.8	± 4.1	10.6	± 3.6
Widower	53.6	± 11	13.2	± 4.8	9.4	± 3.7
Type of living						
Interior flat	63.4	± 11.1	15.4	± 3.7	11.4	± 3.8
Exterior flat	61.8	± 10.7	14.6	± 3.9	10.4	± 3.6
Semidetached house	62	± 10.6	15.1	± 3.8	10.8	± 3.6
Particular house	60.7	± 10.2	14.3	± 4	10.2	± 3.7
Category						
University (nurse)	61.8	± 10.9	14.5	± 3.9	10.4	± 3.7
Not university (nurse auxiliary)	62.2	± 10.5	15	± 3.8	10.8	± 3.6
Work experience						
−1 year	64.9	± 9.4	15.5	± 3.2	11.6	± 3.5
1−5 years	64.4	± 9.9	15.5	± 3.4	11.1	± 3.6
5−10 years	64.4	± 9.4	15.5	± 3.6	11	± 3.7
10−15 years	64.1	± 10.8	14.8	± 3.8	10.8	± 3.9
>15 years	59.3	± 10.9	14	± 4.1	10.1	± 3.6

**TABLE 3:** Univariate and multivariate regression model

	Univariate regression model									Multivariate regression model								
	Stressors			Perceived emotions			Coping strategies			Stressors			Perceived emotions			Coping strategies		
	C	95% CI^†^	*p‐*value	C	95% CI^†^	*p‐*value	C	95% CI^†^	*p‐*value	C	95% CI^†^	*p‐*value	C	95% CI^†^	*p‐*value	C	95% CI^†^	*p‐*value
Sex																		
Male*																		
Female	2.4	0.6–4.2	0.010	1.3	0.6–1.9	<0.001				2.5	0.8–4.3	0.004	1.3	0.6–1.9	<0.001			
Age																		
<30*																		
30−45	−1.8	−3.3 to −0.2	0.022	−0.8	−1.4 to −0.3	0.003				−0.4	−2.4 to 1.6	0.687	−0.5	−1.2 to 0.2	0.193			
>45	−5.7	−7.2 to −4.1	<0.0001	−1.6	−2.2 to −1.1	<0.001	−1.1	−1.6 to −0.5	<0.001	−3	−5.2 to −0.7	0.01	−1.2	−2 to −0.3	0.006			
Marital status																		
Single*																		
Married	−1.8	−3.1 to −0.6	0.004	−0.7	−1.2 to −0.3	0.002	−0.8	−1.3 to −0.4	<0.001	0.8	−0.7 to 2.2	0.293						
Divorced	−4.7	−6.9 to −2.5	<0.001							−1.9	−4.3 to 0.4	0.102						
Widower	−9.8	−14.6 to −5.1	<0.001	−1.9	−3.6 to −0.2	0.028	−1.7	−2.2 to −0.2	0.047	−5.9	−10.7 to −1.2	0.015						
Type of living																		
Interior flat*																		
Exterior flat				−0.8	−1.5 to −0.2	0.007	−1	−1.6 to −0.4	<0.001				−0.5	−1.1 to 0.1	0.103	−0.8	−1.3 to −0.2	0.008
Semidetached house													0.2	−0.6 to 1	0.587	−0.2	−1 to 0.5	0.576
Particular house	−2.7	−4.9 to −0.5	0.016	−1.1	−1.9 to −0.3	0.006	−1.3	−2 to −0.5	0.001				−0.8	−1.6 to −0.1	0.047	−1	−1.8 to −0.3	0.008
Category																		
University nurse*																		
Not university nurse auxiliary				0.5	0.1−0.9	0.011							0.6	0.2−1	0.006			
Work experience																		
<1 year*																		
1−5 year										−0.6	−3.6 to 2.5	0.718				−0.5	−1.5 to 0.6	0.39
5−10 year										−0.5	−3.7 to 2.6	0.737				−0.5	−1.6 to 0.6	0.376
10−15 year										−0.6	−4 to 2.7	0.714				−0.7	−1.9 to 0.5	0.260
>15 year	−5.6	−8.5 to −2.6	<0.001	−1.4	−2.5 to −0.4	0.008	−1.5	−2.5 to −0.4	0.004	−4.5	−7.6 to −1.3	0.006				−1.1	−2.2 to 0	0.049

* Reference; †CI: confidence interval.

### Relationship between sociodemographic variables and dimensions, stressors, perceived emotions, and coping strategies

The analysis of the sex variable in relation to study dimensions (stressors, perceived emotions, and coping strategies) shows a mean for the stressors of 62.2 ± 10.5 in women and 59.8 ± 12.5 in men. A value of p = 0.010 was estimated, and these differences were statistically significant. We observed significance (p < 0.001) with respect to the mean of emotions perceived in women (14.9 ± 3.8) and men (13.6 ± 4.4).

Age presented statistically significant differences in the dimension of stressors, perceived emotions, and coping strategies. Age is a protective factor for all dimensions, stressors, perceived emotions, and coping strategies; a p‐value of < 0.001 was estimated for three dimensions in people over 45 years old and coefficients of −5.7 for stressors, −1.6 for perceived emotions, and −1.1 for coping strategies.

Marital status presented statistically significant differences among the three dimensions, with single being a risk factor as compared with other statuses. The widowed marital status shows a coefficient of −9.8 and a p‐value of < 0.001 for stressors. Analysis of the type of living indicates that interior floors are risk factors for stressors, perceived emotions, and coping ability. The particular house shows differences statistically significant for all dimensions, with a protector coefficient of −2.7 for stressors, −1.1 for perceived emotions, and −1.3 for coping. The exterior floors are also protective factors for perceived emotions and coping, showing statistically significant differences. The variable that corresponded to the type of housing was statistically significant for perceived emotions and coping strategies.

In addition, with these results, multivariate regression (Table 3) shows that the sex and age variables showed statistically significant differences concerning stressors and perceived emotions.

### Relationship between category variables and the dimensions, stressors, perceived emotions, and coping strategies

The professional category variable presented statistically significant differences for perceived emotions. With respect to the analysis of the dimensions and the variable “type of employment relationship,” an average of perceived emotions of 14.5 ± 3.9 for university (nurse) and 15 ± 3.8 for nonuniversity (auxiliary) was observed, with a p‐value of 0.011. These differences were statistically significant.

The length of work experience showed statistically significant differences; in professionals with experience over 15 years, a protector coefficient of −5.6 for stressors, −1.4 for perceived emotions, and −1.5 for coping strategies was observed, so that the time of experience showed statistically significant differences in stressors and coping strategies.

## DISCUSSION

In this study, the multivariate analysis confirmed that being older, not being single, living in an independent house, and having more than 15 years of work experience protected against stressors and perceived emotions, and were associated with a greater use of coping techniques. Being male was also protective against stressors and threatening emotions, although no statistically significant differences were observed between women and men in the use of coping strategies. The results for nursing assistants as compared with university nurses showed that nursing assistants report a greater overload of negative emotions. Spanish nurses are subjected to high levels of stress and negative emotions that had impacted their mental health. Some studies have found the same effect in Portuguese nurses (Sampaio et al., 2021) or Chinese nurses (Hu et al., 2020; Ornell et al., 2020). This psychological stress can be the cause of the development of health problems such as depression, impotence, stress (Ornell et al., 2020), anguish (Chew et al., 2020), anxiety (Paiano et al., 2020; Rajkumar, 2020), sleep disorders, irritability, anger, denial, fear insomnia (Moreira et al., 2020), burnout syndrome, posttraumatic stress (Ramírez‐Ortiz et al., 2020), and even suicide risk (Reger et al., 2020). Exceeding psychological and emotional limits of healthcare professionals increases the risk of psychological stress, with an increased likelihood of developing these mental disorders, which can be observed in the long term (Almaguer et al., 2020).

Regarding this, being a nurse and being a woman are two factors strongly associated with a negative experience during the COVID‐19 pandemic that leads to more severe symptoms of mental health deterioration (Alexander & Klein, 2001; Kessler et al., 1994; Lai et al., 2020). Several studies have indicated that women are more prone than men to internalize the trauma they experience, resulting in more psychological symptoms (Huang et al., 2020; Neitzke, 2016). Studies explain this fact as a consequence of the caregiving role of female nurses, both at work and at home. It is highly probable that this greater experience of stressful situations among female nurses is related to their habitual dual role as caregivers in the workplace and in the family. In this sense, fear is a natural human adaptive response to threatening situations, and SARS‐CoV‐2 has been experienced as a threat. Therefore, we can consider this increased vulnerability of women for fear of their family members becoming infected to be logical (Crowe et al., 2021; Liu et al., 2020a), especially if they have children.

Older age and longer professional experience are associated with lower psychological impact (stressors and emotions) and greater use of coping strategies, as supported by other studies (Almaguer et al., 2020; Zhang et al., 2021). Younger nurses tend to have more stress, which is related to less working experience, less adaptation, and greater vulnerability to a volatile and overloaded healthcare environment (Sampaio et al., 2021). In Tang et al. (2017), after investigating anxiety levels in nurses following the H7N9 outbreak, anxiety levels were found to be higher among younger participants and associated with a decrease in knowledge, skills, and professional experience. In addition, older age, job consolidation, and experience result in the development of coping strategies that reduce stress, impact of stressors, and emotional overload (Almaguer et al., 2020; Torales et al., 2020; Zhu et al., 2020). This fact can be perfectly explained by the Patricia Benner theory, which maintains that nurses, through experience, evolve from beginners to experts, and this allows them to develop skills and face complex clinical situations with greater solvency (Izquierdo Machín et al., 2016).

Single nurses have a higher risk of psychological impact than those who are married, divorced, or widowed; it has probably been the unmarried, as well as younger people, who have moved and suffered the most from isolation from their physical, familial, and social environment (Zhang et al., 2021).

Two levels of nursing professionals exist in Spain, i.e., nurses who have a university degree and nursing assistants with a nonuniversity degree. We found a greater impact of perceived negative emotions among auxiliary nurses than among university graduate nurses. Although it has been shown that more curricular development is needed to deal with crisis situations (Aznar et al., 2020), we think that this fact can be explained by the greater and more extensive academic preparation of graduate nurses for incompetencies associated with stress and emotion management in emergency and critical situations.

### Strengths and limitations of the study

This study gathered a large sample of nurses from all over Spain, which adjusts to the heterogeneity of Spanish nurses in relation to the proportion of men and women, professional categories, and ages (Meseguer Gancedo, 2018), which allows us to assume that the high levels of stress and emotional overload identified in this study and the poor use of coping strategies are generalizable across the Spanish nursing profession. Although the stratification of the data is consistent with the population data, the present study used nonprobability sampling. In order to generalize our results, future longitudinal studies should be conducted using probability sampling. Despite the results of interest provided by this research work, it would be convenient to continue deepening the subject of the study with experimental research that specifically relates the mental health of nurses in continuous contact with patients infected by SARS‐CoV‐2 and that does not include the limitations of cross‐sectional studies.

## IMPLICATIONS FOR NURSING AND/OR HEALTH POLICY

Few studies have been conducted in the country with a large sample like the present research. In this study, we try to analyze the psychological impact suffered and determine risk factors and target population. It facilitates the design of preventive procedures and coping strategies to guarantee better mental health of professionals, less absenteeism, better stress control, and less burnout, and, in short, nurses with more general resources of resistance in borderline situations to provide higher quality care.

## CONCLUSIONS

This study suggests an important psychological impact among Spanish nurses. The female nurses who are younger, have less work experience, have not built a family of their own, and live in smaller or indoor flats may be more vulnerable to the effects of the COVID‐19 pandemic on their mental health. We should not wait for a new pandemic to design and adopt effective strategies and measures for the protection of nurses' mental health, as well as for the prevention and early diagnosis of possible mental health problems.

## FUNDING STATEMENT AND INSTITUTIONAL REVIEW BOARD STATEMENT

The survey was anonymous and did not collect personal data or devices that could identify the informant. The information was treated confidentially and anonymously since it was dissociated data, following the Data Protection Regulation (EU) 2016/679 of the European Parliament and the Spanish Organic Law 3/2018. The study was approved by the ethics committee of the Rioja Biomedical Research Center (CIBIR) with protocol reference CEImLar P.I. 416). This study has not received any funding.

## CONFLICTS OF INTEREST

The authors declare no conflict of interest. The researchers did not declare any type of ethical, moral, or legal conflict, nor did they claim to have received financial compensation of any kind. The participants did not receive any type of compensation for answering the questionnaire, as it was voluntary.

## AUTHOR CONTRIBUTIONS

Conceptualization: Pablo del Pozo‐Herce and Rebeca Garrido Garcia; methodology: Raúl Juarez Vela; validation: Iván Santolalla‐Arnedo and Francisco José Rodriguez Velasco; formal analysis: Raúl Juarez Vela and Michał Czapla; investigation: Emmanuel Echániz‐Serrano and Iván Santolalla‐Arnedo; data curation: Guadalupe Gil‐Fernandez and Regina Ruiz de Viñaspre‐Hernandez; writing—original draft preparation: Vicente Gea‐Caballero and Regina Ruiz de Viñaspre‐Hernandez; funding acquisition: Francisco José Rodriguez Velasco and Raúl Juarez Vela; all the authors have read and agreed to the final version of the manuscript.

## PERMISSIONS TO USE, MODIFY, OR TRANSLATE SCALES IN THE RESEARCH PROCESS

The questionnaire used was created by the authors of this article.

## POPULATION AND SCOPE OF THE STUDY

The study was developed through the platform www.estudioenfermeria.com to obtain layered data and access control of the sample.

## Supporting information

Supporting Information

## Data Availability

The data are available by contacting the corresponding author.
