# Supplementary material for: Psychological impact on care professionals due to the SARS‐Cov‐2 virus in Spain
Source: Int Nurs Rev. 2022 Feb 2;69(4):520–8. doi: 10.1111/inr.12748 (PMC9790592; doi:10.1111/inr.12748)
Supplement: Supplementary file 1 — Supporting Information [file INR-69-520-s001.docx]

**Suplementary 1.** Score dimensions: **(a)** Stressors; **(b)** perceived emotions; **(c)** coping strategies

(a)

(b)

(c)
